# A New Approach for Explaining and Treating Dry Sockets: A Pilot Retrospective Study

**DOI:** 10.7759/cureus.41347

**Published:** 2023-07-04

**Authors:** Wael Khalil

**Affiliations:** 1 Oral and Maxillofacial Surgery, Faculty of Dental Medicine, Lebanese University, Beirut, LBN

**Keywords:** bacterial biofilm, enterococcus faecalis, pseudomonas aeruginosa, alveolar osteitis, localized alveolitis, dry socket

## Abstract

Objective

Dry socket, a common complication following a tooth extraction, is characterized by severe and radiating pain that typically begins one to four days after the extraction. Despite several risk factors, the exact cause and underlying mechanisms of dry sockets remain unclear. This study aims to propose a novel pathogenesis and management approach for dry sockets based on an infectious process.

Methods

The study was conducted by reviewing medical records, at a private dental clinic, of patients who fit the inclusion criteria; these patients appeared to have come between April 2022 and April 2023. The study included all patients with age ≥17 years diagnosed with dry socket that was resistant to conventional topical treatment, and who received treatment with ciprofloxacin 500 mg three times per day during the study period.

Results

Out of 15 patients who received treatment with ciprofloxacin 500 mg three times per day during the study period, 11 patients (73.3%) were completely relieved of symptoms within 24 hours, with no need for additional painkillers or nonsteroidal anti-inflammatory drugs (NSAIDs). In addition, two patients (13.3%) had a partial response after 48 hours, where their pain was ameliorated from severe to moderate with the use of conventional painkillers (including paracetamol and NSAIDs) and steroidal anti-inflammatory drugs such as dexamethasone (8 mg IM daily) to have total relief. On the other hand, the other two patients (13.3%) had a negative response to the treatment and were out of reach for follow-up.

Conclusion

These clinical outcomes, coupled with previous laboratory data, could explain all clinical aspects of dry sockets and provide substantial support for the hypothesis that an infectious mechanism plays the principal role in the pathophysiology of dry sockets.

## Introduction

Dry socket is recognized as the most common complication that occurs after a dental extraction. While it is estimated to have an incidence rate of around 3%, it can escalate up to 30%, specifically in cases involving the surgical removal of mandibular wisdom teeth [1]. The onset of dry socket typically occurs within one to four days after the extraction, marked by the partial or complete disintegration of the blood clot and the subsequent exposure of the alveolus. Clinically, the alveolus becomes sensitive to touch, often appearing empty and denuded. However, it may be covered by a grayish tissue layer in some instances. Additional symptoms, such as lymphadenitis and halitosis, may also be present in certain cases.

The pain associated with dry sockets is often unresponsive to analgesics and anti-inflammatory drugs, and it can radiate to the ear and neck. The existing literature suggests that the main cause of dry sockets is the absence, abnormal formation, or early disintegration of the blood clot in the socket following tooth extraction [2]. Various local and systemic factors have been identified as predisposing factors. Smoking is commonly cited as a significant factor contributing to dry socket occurrence, along with other factors such as traumatic extraction, using vasoconstrictors such as epinephrine, the amount of anesthesia administered, pre-existing infections, non-compliant patient, and poor oral hygiene [3,4]. Systemic conditions such as age, gender, diabetes, chemotherapy, the use of oral contraceptives, and anti-inflammatory drugs have also been associated with an increased incidence of dry sockets [5].

In terms of local measures and management, the literature suggests several preventive strategies to reduce the occurrence of dry sockets, including local hemostatic agents, gelatin sponges, plasma rich in growth factors, and laser application [4,6,7]. Treatment options for dry sockets involve the removal of debris from the socket using 0.2% chlorhexidine or saline. Additionally, sedatives such as eugenol may be applied to alleviate pain [8]. The use of antibiotics as a preventive measure has shown efficacy. However, its use as a curative measure when prescribed systemically remains debatable [4,9].

This research’s main aim is to propose a new paradigm for explaining and treating dry sockets based on an infectious process by presenting 15 cases of dry socket rebellion to topical treatment that were treated with ciprofloxacin based on previous findings of bacterial cultures and antibiograms to explore a new microbiological aspect of dry sockets [10,11].

## Materials and methods

Study design

This pilot retrospective study aimed to suggest a new pathogenesis of dry sockets based on an infectious process by investigating the effect of an antimicrobial agent, which is ciprofloxacin, on patients with rebellion dry socket cases. The study was conducted by reviewing medical records at the practitioner's private dental clinic for patients who fit the inclusion criteria; these patients appeared to have come between April 2022 and April 2023.

The study included all patients aged ≥17 years diagnosed with dry sockets resistant to conventional topical treatment, and who received treatment with ciprofloxacin 500 mg three times per day during the study period. A total of 6 out of the 15 included patients were smokers (40%), 8 had surgical extraction done (53.3%), and 13 (86.6%) had a previous infection before extraction (pericoronitis, 46.6%; periodontitis, 6.6%; periapical infection, 33.3%). Patients with incomplete medical records or those who were out of reach for signing the consent form were excluded from the research. Patients included were recalled to sign a consent form for approval of usage of their data in this research.

Data collection

A comprehensive review of electronic medical records was performed to collect relevant data. The following information was extracted for each patient: demographics (age, gender), number of teeth extracted, type of extraction (surgical, simple), presence or absence of previous infection before extraction, medical history, oral hygiene condition, smoking or non-smoking state, and any other note that could be added to the case. Patient confidentiality was strictly maintained, and data were anonymized and securely stored throughout the study. The details of all the included cases are presented in Table 1.

**Table 1:** Details of cases included in the study ASA, American Society of Anesthesiologists ASA1: healthy, non-smoking, no or minimal alcohol-use patients; ASA3: patients with severe systemic disease *Good oral hygiene was considered when dental plaque was absent from the tooth surfaces. Bad oral hygiene was considered when dental plaque was present on tooth surfaces. *Heavy smokers were considered patients who smoked more than 20 cigarettes per day. Moderate smokers were considered patients who smoked fewer than 20 cigarettes per day.

Case	Tooth number	Age	Gender	Simple extraction	Surgical extraction	Previous infection	Medical history	Oral hygiene	Smoking status	Notes
1	46	60	Female		X	+ Periodontitis	ASA1	Good*	Non	
2	48	23	Male	X		-	ASA1	Poor (bad)	Non	
3	35	38	Female	X		+ Periapical infection	ASA1	Poor (bad)	Heavy*	History of previous dry sockets
4	14	55	Female	X		+ Periapical infection	ASA1	Good	Non	
5	48	65	Male		X	+ Pericoronitis	ASA1	Good	Heavy	
6	38	18	Female		X	+ Pericoronitis	ASA1	Poor (bad)	Heavy	
7	34	45	Male	X		+ Periapical infection	ASA1	Good	Non	
8	38	25	Male		X	-	ASA1	Poor (bad)	Non	
9	38	17	Female		X	+ Pericoronitis	ASA1	Good	Non	
10	48	21	Female		X	+ Pericoronitis	ASA1	Good	Moderate	
11	38	24	Male		X	+ Pericoronitis	ASA1	Poor (bad)	Heavy	
12	38	40	Male	X		+ Pericoronitis	ASA1	Poor (bad)	Heavy	
13	48	17	Female		X	+ Pericoronitis	ASA1	Good	Non	
14	46	55	Male	X		+ Periapical infection	ASA3	Poor (bad)	Non	
15	35	40	Female	X		+ Periapical infection	ASA1	Good	Non	

The common clinical features recorded in the included patients' files were (1) the onset of symptoms between one and four days, (2) the clinical appearance of the denuded socket, (3) the unresponsive aspect of pain to conventional analgesics such as pure analgesics and nonsteroidal anti-inflammatory drugs (NSAIDs), and (4) the rebellious aspect of the conventional topical treatment of dry sockets.

## Results

A total of 15 patients were included in the retrospective analysis, with a mean age of 41 years (range, 17-65 years). Among the participants, 46.6% were male and 53.4% were female; 46.6% of them had surgical extraction, while 40% were smokers.

The primary outcome measure of the study was the response of patients with rebellion dry sockets to ciprofloxacin. Out of 15 patients who received treatment with ciprofloxacin 500 mg three times per day during the study period, 11 patients (73.3%) were completely relieved of symptoms within 24 hours, with no need for additional painkillers or NSAIDs. In addition, two patients (13.3%) had a partial response after 48 hours, where their pain was ameliorated from severe to moderate with the use of conventional painkillers and steroidal anti-inflammatory drugs such as dexamethasone (8 mg IM daily) to have total relief. On the other hand, the other two patients (13.3%) had a negative response to the treatment.

**Figure 1:**
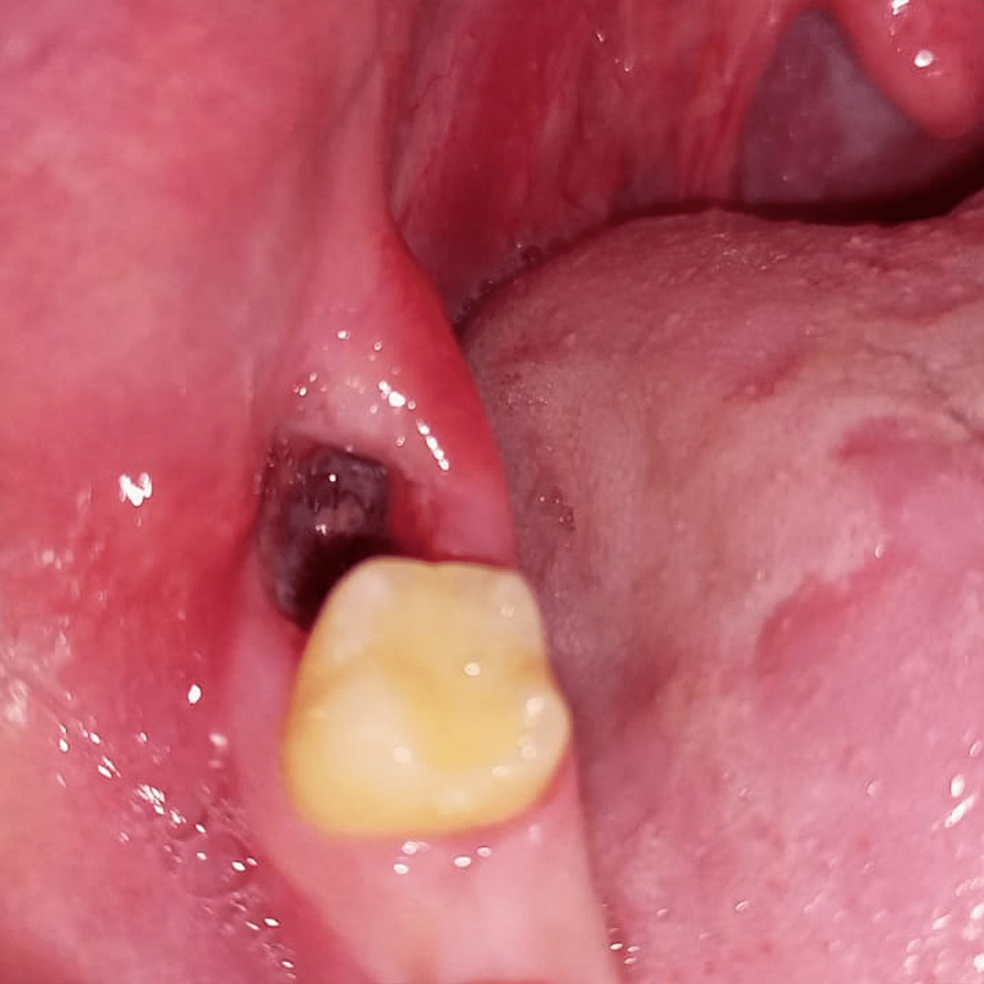
Clinical presentation of the dry socket that occurred in a 17-year-old female after extraction of her lower right third molar

**Figure 2:**
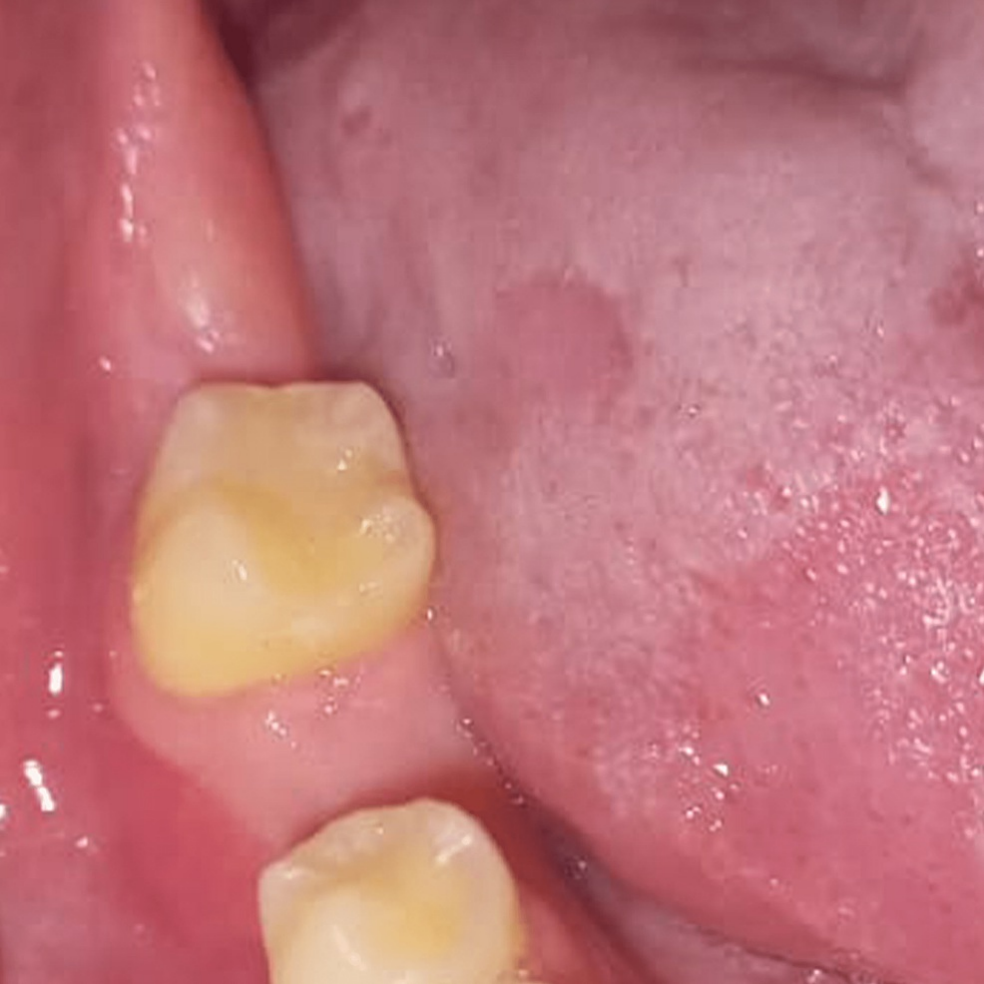
Clinical presentation of the soft tissues of the lower right posterior region after healing of the dry socket

Here, we have included a case of a 17-year-old female who underwent surgical extraction of the lower right third molar. The records of the patient showed a complete relief of symptoms within 24 hours. Figure 1 shows the clinical condition after the occurrence of the dry socket and Figure 2 shows the condition after recovery.

## Discussion

In this pilot retrospective study, patients with dry sockets experienced either total (11 patients) or partial relief (two patients) after receiving ciprofloxacin 500 mg three times per day. While the two patients who had negative responses to ciprofloxacin were out of reach for follow-up, their experience can be attributed to either a misdiagnosis of their case or bacteria resistant to ciprofloxacin. These bacteria might be *Pseudomonas aeruginosa*, *Enterococcus faecalis*, or other bacteria having similar properties [10,11]. The positive response to ciprofloxacin supports the hypothesis of an infectious process behind the dry socket.

Despite extensive research, the exact cause of dry sockets has not been definitively identified. However, various local and systemic risk factors have been associated with their occurrence. The most important risk factor is smoking, which has a double impact on local and systemic levels. Local factors include challenging or traumatic extractions, the use of vasoconstrictors such as epinephrine, the amount of anesthesia administered, pre-existing infections such as pericoronitis and periodontitis, and inadequate oral hygiene [1]. Systemic factors include age, the use of oral contraceptives and anti-inflammatory drugs, and the presence of comorbidities such as diabetes and chemotherapy [1,2]. Women are at a higher risk, and dental extraction performed within the first three weeks of the menstrual cycle may further increase the incidence of dry sockets [4]. In this study, 6 out of 15 patients with dry sockets were smokers, while the non-smokers (9 out of 15) were having at least one other risk factor for dry sockets, which was either surgical extraction, previous infection, or poor oral hygiene. More specifically, five out of nine patients had two combined risk factors from the aforementioned ones.

Current theories regarding the pathophysiology of dry sockets revolve around the role of blood clots, suggesting that they may be caused by a lack of clot formation, abnormal clot formation, or early clot disintegration [1,4]. In this context, Tolstunov’s research revealed that effective management of bleeding in the socket after tooth extraction plays a crucial role in ensuring a successful and uncomplicated healing process [12]. Additionally, Noroozi and Philbert argued that fibrinolytic activity is believed to have an impact on the stability and structure of the blood clot formed after tooth extraction [13]. The same theory was explained in Gowda et al.’s research, which showed that the active fibrinolytic activity after extraction caused the disintegration of the blood clot [5]. It is important to mention that most of the studies have focused on risk factors leading mainly to the disintegration of the blood clot, such as smoking, physical dislodgement of the clot, bacterial infection, and excessive irrigation or curettage of the socket [4,5].

Bacteria have never been identified as the main or principal cause of dry sockets in the available literature on dry sockets. However, they have been implicated as a contributing factor in the development of dry sockets as they impair the socket’s healing by fibrinolysis. In addition, *Treponema denticola*, a bacterium commonly found in the oral cavity, has been associated with the release of enzymes such as streptokinase and staphylokinase, which can activate fibrinolysis and impact clot formation [4]. Other bacteria, including *Capnocytophaga ochracea*, *Fusobacterium nucleatum*, *Prevotella melaninogenica*, *Streptococcus anginosus*, *Treponema socranskii*, and *Streptococcus sanguis*, can also affect the alveolar repair process by inducing higher levels of C-reactive proteins [5,14-16].

It is worth noting that while bacteria forming biofilms, such as *P. aeruginosa* and *E.* *faecalis*, have not been identified as the primary cause of dry sockets, they can contribute to bacterial resistance and potentially hinder the effectiveness of some systemic antimicrobial treatments [14]. *P. aeruginosa* can disseminate into the bone and has been associated with mandibular osteomyelitis [6,7]. These bacteria, along with other bacterial types having the same properties, are usually resistant to antimicrobial molecules that are usually prescribed in dental practice, such as penicillin A. Thus, the inefficiency of such antibiotics in patients with dry sockets was the reason for several authors’ exclusion of an infectious process as the cause of this complication. However, the actual cause could have been bacterial resistance against those antibiotics, which would normally appear during an infectious process.

Based on a previous research, which involved positive bacterial cultures in four swabs out of six (*P. aeruginosa* and *E. faecalis*) [11], and the present study's findings showing a positive response in more than 86% of dry socket cases treated by ciprofloxacin, one can reach a conclusion that the pathogenesis of dry sockets cannot be solely attributed to a disorder in blood clot formation. The authors hypothesized that dry socket is a complication that may be caused by a specific bacterial infection, and the disorder in blood clot formation is considered a stage in the pathophysiological infectious process. This hypothesis can be supported by multiple arguments and facts.

There are three arguments concerning this issue. The first argument suggests that blood clot disorders do not explain all the clinical aspects of dry sockets. They can explain the delayed onset and the exposed alveolus seen in dry sockets, but cannot explain the severity of pain and lymphadenitis whereas the infectious process can better explain the pathophysiology of dry socket and its associated clinical manifestations, which include the following four hallmarks: the onset of clinical symptoms that aligns with the incubation time of some bacteria [8], the exposed and denuded alveolus due to the fibrinolytic action of *P. aeruginosa* or other potentially involved bacteria [9,10,15], the inflammation process and severity of pain that occurs due to the activation of several inflammatory pathways by multiple bacteria including *P. aeruginosa *and *E. faecalis* [16,17], and finally, the last hallmark, recurrent lymphadenitis that is usually a sign of an infectious process in the nearby area.

The second major supporting argument is that all the predisposing factors previously mentioned, which are known to contribute to the development of dry sockets, are actually associated with a decrease in local and systemic immune function and dysregulation of polymorphonuclear neutrophils (PMNs). For instance, smoking has been shown to have a negative impact on oral PMNs and local immunity [3]. Furthermore, the presence of a pre-existing infection at the extraction site can impair the function of PMNs. Women who take oral contraceptives have been found to have a lower phagocytic capacity in their neutrophils compared to those who do not [18]. Additionally, the trauma during tooth removal can induce ischemia at the site, and difficult and traumatic procedures can elevate the patient's stress levels, potentially leading to neutrophil dysregulation [19].

The final argument that supports this research's hypothesis is that existing theories that rely on the role of blood clots are insufficient to explain the development of dry sockets as a complication of coronectomy. A coronectomy is a dental procedure where only the crown of the tooth is removed, leaving the root intact in the socket. In this procedure, there is no complete socket formation or presence of a blood clot, which challenges the explanation solely based on blood clot involvement [20].

Now concerning the facts supporting the hypothesis suggested above, we should mention the bacterial culture analysis conducted on dry socket cases that revealed the presence of *P. aeruginosa* and *E. faecalis* [11]. This finding indicates a possible association between *P. aeruginosa*, *E. faecalis*, and other bacteria having similar properties and the occurrence of dry sockets [21,22]. Furthermore, the antibiotic protocol specifically designed to target *P. aeruginosa* and possibly other bacteria, using ciprofloxacin at a dosage of 20 mg/kg/day, has demonstrated high efficacy in the treatment of dry sockets [10]. In contrast, the use of broad-spectrum antibiotics, such as penicillin A and clavulanic acid, which do not specifically target *Pseudomonas*, was found to be ineffective in treating dry sockets [9]. In addition, previous studies have indicated that, among the molecules used for systemic prophylaxis, azithromycin has shown efficacy in reducing the incidence of dry sockets. Azithromycin is the only molecule among the studied options that has demonstrated therapeutic effects, specifically against *P. aeruginosa*, the bacteria associated with dry sockets [1,11]. These findings suggest that azithromycin may be a valuable prophylactic and therapeutic option for preventing and treating dry sockets, particularly in cases where *P. aeruginosa* infection is suspected or confirmed [23].

In the same context, it is important to mention that the isolation of *P. aeruginosa* and *E. faecalis* in dental infections has been reported by several authors, which further supports this study's hypothesis regarding the involvement of this bacterium in the development of dry sockets [15,17,24,25]. It should be noted that *P. aeruginosa* has the potential to be present in the tissues surrounding the tooth even before the extraction takes place, and it might be in a dormant state that would be exacerbated during extraction. Similarly, *E. faecalis* can be present in saliva, causing contamination of the extraction socket [26]. Additionally, during the extraction procedure, the socket can become contaminated with *P. aeruginosa* and *E. faecalis* from other sources, including saliva or irrigating water from the handpiece. Based on our observations and findings, we propose that the presence of bacteria, particularly those capable of forming biofilms, such as *P. aeruginosa* and *E. faecalis*, may explain the underlying pathogenesis of dry sockets [14]. In this context, *P. aeruginosa*, *E. faecalis*, and other bacteria with similar properties can be considered the primary causative agents of this complication. Finally, it is important to mention that the effective application of topical eugenol in the socket, as described by many authors, supports the hypothesis that the bacterial biofilm plays a causal role in dry sockets rather than the theory based solely on the blood clot [2]. Eugenol not only provides sedative effects but also exhibits antibacterial properties and acts as a biofilm disruptor, particularly against *P. aeruginosa* and *E. faecalis* [27].

Considering all that is mentioned above, several management measures for dry sockets are proposed based on the hypothesis suggested in this study. These measures can be implemented pre-operatively by rinsing the socket with a 2% chlorhexidine solution for 30 seconds, per-operatively by irrigating the socket alternately with chlorhexidine 0.2% and iodine 5%, or post-operatively by prescribing the appropriate antibiotic in the case of infection. These measures should focus on reducing the bacterial load to reduce the incidence of infection after surgery.

Limitations

It is important to acknowledge the limitations of this study in order to gain a comprehensive understanding of the findings. The hypothesis is based on previous studies of serial cases with bacterial cultures in a few cases. A larger sample is needed with bacterial cultures; also, microbial exploration should be done for sites other than the socket, as control, such as saliva or a healthy site from oral mucosa. In addition, a prospective study on a larger group is needed with bacterial cultures and antibiograms on all included patients in order to choose the appropriate antibiotic and to assess and evaluate its effect on socket healing and pain relief. Lastly, future studies should focus on discussing controlled variables in order to reveal a significant association between bacteria and dry sockets on one hand and the efficiency of antibiotics in healing and pain relief on the other.

## Conclusions

Dry socket is a highly troublesome complication that can arise following tooth extraction. Its management poses challenges, particularly when conventional treatment methods prove ineffective. The laboratory and clinical findings provide strong evidence that this is caused by an infection. However, it is important to note that the causal bacteria may not be limited to those that have been reported. Finally, understanding the causes of dry sockets is crucial for dental surgeons to reduce their incidence through antimicrobial measures and implement targeted treatment strategies. Further studies should be conducted in order to establish the pathogenesis of dry sockets.
